# Clavicular nutrient foramina in dry human bones: surface distribution, regional localisation, and surgical relevance

**DOI:** 10.1007/s00276-026-03943-6

**Published:** 2026-07-08

**Authors:** Kashish Gupta, Rohini Punja, Arun Kumar Shetty, Purnima Adhikari, Sonal Nayak

**Affiliations:** 1 Department of Anatomy, Kasturba Medical College, Manipal Academy of Higher Education, Manipal, India; 2 Department of Orthopaedics, Alva’s Institute of Medical Sciences and Research Centre, Moodbidri, Karnataka India; 3 Division of Anatomy, Department of Basic Medical Sciences, Manipal Academy of Higher Education, Manipal, India

**Keywords:** Clavicle, Nutrient foramen, Foraminal index, Morphometry, Surgical anatomy

## Abstract

**Purpose:**

Iatrogenic disruption of the clavicular nutrient artery during fracture fixation has been implicated in non-union. Population-specific data on nutrient foramen location are lacking for Indian skeletal collections. This study characterises clavicular nutrient foramina in an institutional dry bone series and benchmarks the findings against published international data.

**Methods:**

121 unpaired dry adult human clavicles (60 right, 61 left) of unknown age and sex were examined. Foramen count, surface location (inferior/posterior/superior/anterior), total clavicular length (TL), distance of the foramen from the sternal end (DNF), and foraminal index (FI = DNF/TL × 100) were recorded. Segment classification (medial/middle/lateral third) was based on FI tertiles (0–33%, 33–66%, 66–100%), following the convention of Hughes [8], which allows comparison across bones of different total lengths. Categorical variables (foramen count, surface distribution) were compared between sides using Fisher’s exact test; continuous variables (TL, DNF, FI) were compared using independent-samples t-tests (*p* < 0.05).

**Results:**

146 foramina were identified; 114 clavicles (94.2%) bore at least one foramen. Single foramina predominated (76.9%; 95% CI 68.6–83.5%); seven clavicles (5.8%) had none. Surface analysis revealed near-equal inferior (48.6%; 95% CI 40.7–56.7%) and posterior (49.3%; 95% CI 41.3–57.3%) distribution. Middle-third localisation was observed in 98.2% of foraminated clavicles (FI 33–66%). Mean TL was 138.5 ± 11.3 mm; mean DNF 72.1 ± 12.1 mm; mean FI 52.1 ± 7.9%. No significant bilateral asymmetry was detected in FI (*p* = 0.728) or DNF (*p* = 0.091).

**Conclusion:**

This institutional dry bone series confirms middle-third predominance and identifies near-equal inferior and posterior surface distribution—a pattern also reported by a second independent South Indian study. These findings suggest that both inferior and posterior surfaces of the clavicular midshaft may warrant surgical awareness in this population. Direct clinical recommendations require validation through vascular or biomechanical studies.

## Introduction

The clavicle is the sole osseous link between the axial skeleton and the shoulder girdle, functioning as a dynamic strut for scapular kinematics and a force-transmitting element for upper limb loads [20]. Its superficial course and curved diaphysis render it susceptible to both direct impact and indirect axial loading [20]. Clavicular fractures are among the most common long bone fractures, comprising 2.6–10% of all fractures across age groups; of these, midshaft fractures account for the majority (69–82%) and are associated with a non-union risk of up to 10% following operative repair [3, 16, 17]. Operative treatment by superior plate osteosynthesis or intramedullary nail fixation is well established for displaced midshaft fractures [3, 9, 16].

Intramedullary vascularity is sustained principally by the nutrient artery, which enters the cortex through the nutrient foramen — a periosteum-rimmed opening admitting the nutrient vessel into the medullary canal [10]. For the clavicle, this vessel most commonly arises from the suprascapular artery and enters the diaphyseal cortex, predominantly through the posterior or inferior surfaces [7, 11], though superior entry has been described in a small proportion of cases [11]. Havet et al. [7], in a cadaveric vascular injection study, demonstrated that the clavicular nutrient artery at its cortical entry is non-redundant: its iatrogenic disruption during plate screw drilling, reaming, or subperiosteal dissection may produce endosteal ischaemia contributing to non-union. Knowledge of the typical surface location and longitudinal position of the foramen has been proposed as relevant to reducing the risk of inadvertent nutrient artery disruption during clavicular surgery [7, 12], though the evidence base is largely anatomical and observational.

Indian skeletal population data on clavicular nutrient foramina remain limited. The sole prior study from an analogous South Indian institutional collection — Murlimanju et al. [14] at Mangalore — examined only 52 clavicles without computing mean FI or providing complete surface mapping. Other available Indian data from Bihar [18], Rajasthan [19], and Central India [5] are small series (50–79 clavicles) lacking one or more of: FI computation, four-surface classification, or segment localisation. At *n* = 121, the present series exceeds all prior Indian institutional dry bone clavicular studies in sample size: Murlimanju et al. [14] (*n* = 52), Aggarwal and Ghorai [1] (*n* = 79), Sinha et al. [19] (*n* = 50), Dakshayani and Shivanal [5] (*n* = 50), and Ratnesh et al. [18] (*n* = 50) (Table 4).

Despite systematic review-level evidence [6] identifying methodological deficiencies in existing studies, Indian population-specific data using complete methodology — fine-wire patency confirmation, four-surface reporting, and FI computation — remain absent. The present study provides a methodologically transparent characterisation in this population, with systematic international benchmarking.

## Materials and methods

### Study design and ethics

This cross-sectional morphometric study was conducted on dry human clavicles from the institutional osteological collection of the Department of Anatomy of a South Indian tertiary medical institution. Ethics approval was obtained from the authors’ Institutional Ethics Committee [IEC 258/2025]; although the study used only anonymised dry cadaveric bone specimens, ethics approval was sought as required by institutional policy, and all procedures were conducted in compliance with the conditions of approval.

### Specimen selection

All clavicles in the institutional osteological collection (*n* = 134) were inspected. Thirteen specimens were excluded: 6 with visible fracture lines or callus, 3 with surgical hardware, 2 with evidence of neoplastic or destructive lesions, and 2 with advanced degenerative erosion compromising cortical integrity. Osteomyelitic change was identified macroscopically by the presence of cortical perforation, cloacae, or irregular periosteal reaction inconsistent with the nutrient foramen morphology; such specimens were excluded. All 121 remaining specimens met the inclusion criteria and were examined.

The clavicles were unpaired; no matched right–left pairs from the same individual were available in the institutional collection. Accordingly, no paired analyses were conducted and left–right comparisons were performed using independent-samples tests. Sex and age of specimens were not recorded in institutional records, consistent with most published institutional dry bone series [1, 2, 14, 21, 22].

### Measurement protocol and observer reliability

Two trained observers independently assessed a randomly selected subset of 30 clavicles prior to full data collection, without knowledge of each other’s measurements. K.G., an undergraduate medical student, served as primary observer and P.A., a postgraduate MSc Anatomy student, served as the second independent observer for the reliability subset. For intra-observer reliability, K.G. re-measured the same 30 clavicles after a minimum interval of two weeks, with the original measurements concealed.

Intraclass correlation coefficients (ICC, two-way mixed, absolute agreement) were: TL inter-observer 0.96 (95% CI 0.92–0.98), DNF inter-observer 0.94 (95% CI 0.89–0.97); TL intra-observer 0.97 (95% CI 0.94–0.99), DNF intra-observer 0.95 (95% CI 0.91–0.98). Full data collection was performed by K.G. following confirmation of ICC ≥ 0.90. ICC values are also presented in the Results section.

### Measured parameters

**Fig. 1:**
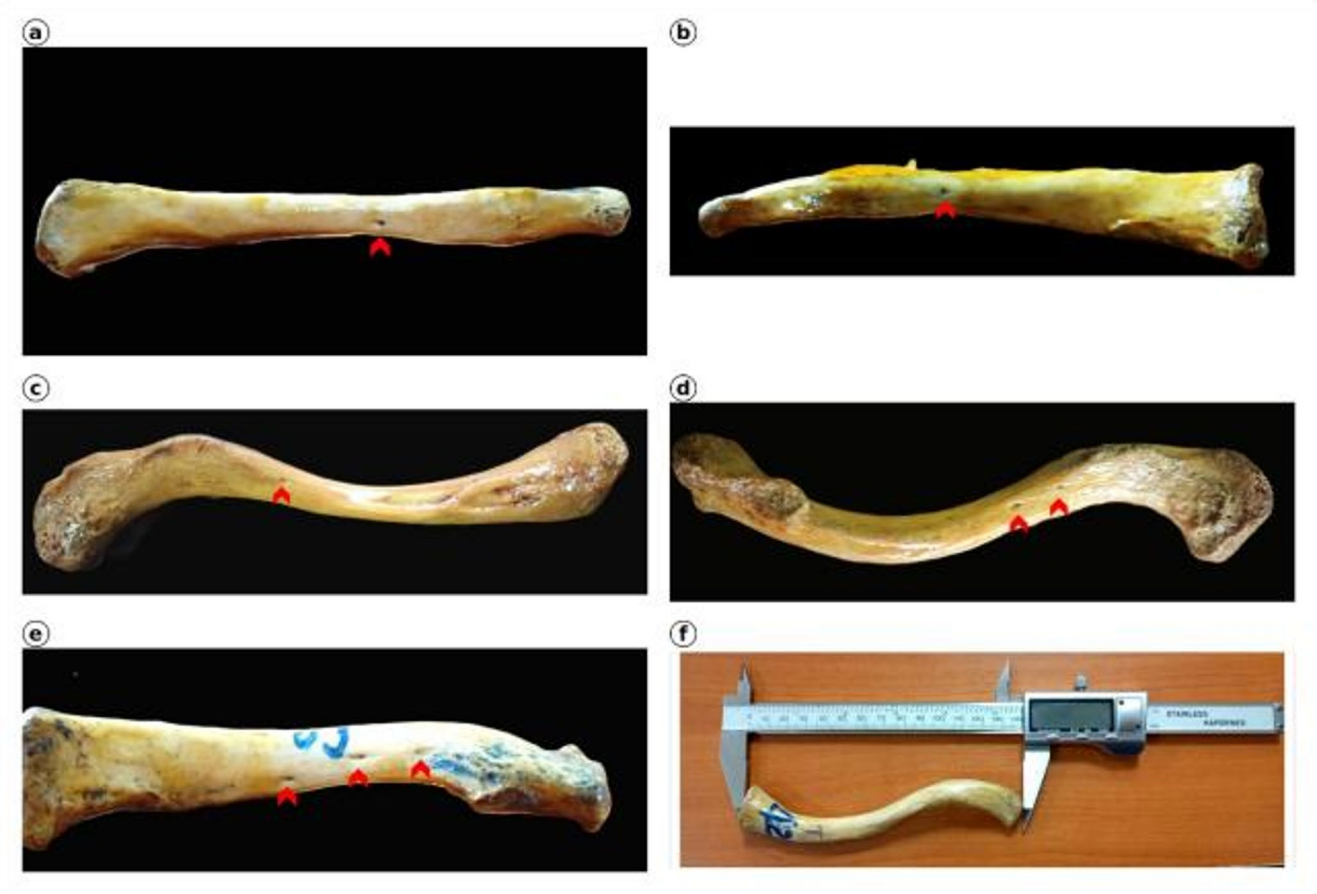
(**a**), (**b**) Posteriorly located single nutrient foramen on the posterior surface of the clavicular midshaft. (**c**) Inferiorly located single nutrient foramen on the inferior surface of the clavicular midshaft. (**d**) Double nutrient foramina on the inferior surface of the clavicular midshaft. (**e**) Triple nutrient foramina (arrowheads) demonstrating the multi-foramen configuration. (**f**) Total clavicular length (TL) measurement using a digital Vernier calliper (precision 0.01 mm). Red arrowheads indicate nutrient foramina in all panels

**Fig. 2:**
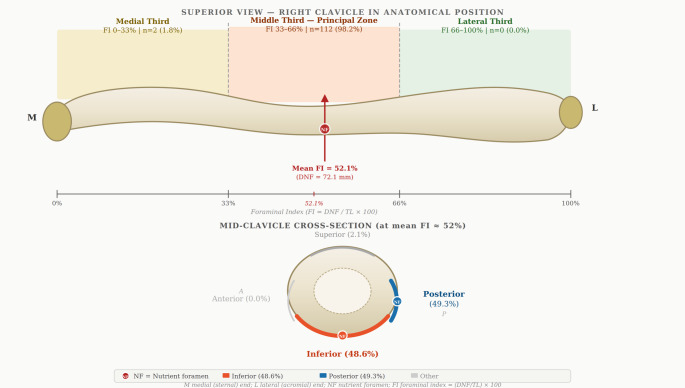
Schematic diagram of the right clavicle in anatomical position showing the foraminal index (FI) zone classification. Prepared by the authors for this study, based on the foraminal index classification introduced by Hughes [8]; zone boundaries follow the tertile division applied in clavicular morphometric studies [6, 12]. The three zones are depicted with equal proportions reflecting equal FI ranges; absolute bone lengths within each zone will vary with total specimen TL. The medial third (FI 0–33%), middle third (FI 33–66%), and lateral third (FI 66–100%) are demarcated. The mean FI of 52.1% is indicated by a red arrowhead. The mid-clavicle cross-section at FI ≈ 52% illustrates the near-equal inferior (48.6%; orange arc) and posterior (49.3%; blue arc) surface distribution. M medial (sternal) end, L lateral (acromial) end, NF nutrient foramen, FI foraminal index = (DNF/TL) × 100

Five parameters were recorded for each specimen:

1. Number of nutrient foramina: openings admitting a fine wire (0.5 mm) into the medullary canal (Fig. 1b and c); minor vascular pits not admitting the wire were excluded.
2. Surface location: each foramen was assigned to the inferior, posterior, superior, or anterior surface.
3. TL: maximum straight-line distance from the medial-most point of the sternal articular surface to the lateral-most point of the acromial articular surface, measured with a digital Vernier calliper (precision 0.01 mm; Fig. 1d).
4. DNF: straight-line distance from the medial-most point of the sternal articular surface to the centre of each identified foramen. For multi-foramen clavicles, DNF of the dominant foramen was used for FI calculation, following Leschinger et al. [12]. Dominance was defined as the largest foramen by external orifice diameter measured with the Vernier calliper. Where two foramina were of equal diameter, the more medially positioned foramen was designated dominant. In all 27 multi-foramen clavicles, a single foramen was clearly larger than the others; the tie-breaking rule was not applied in practice.
5. FI: computed as FI = (DNF/TL) × 100 [8]. Segment classification followed FI tertiles: FI ≥ 0% and < 33% = medial third; FI ≥ 33% and ≤ 66% = middle third; FI > 66% and ≤ 100% = lateral third (Fig. 2). A foramen with FI exactly at 33% is assigned to the middle third; one with FI exactly at 66% is assigned to the middle third. No foramen in the present series had an FI at these exact boundary values.

The present study assessed foramen count, surface location, and FI-based regional localisation. Foramen orifice diameter and canal direction were not assessed, as the primary focus was surface distribution and regional localisation; these represent limitations acknowledged below.

### Statistical analysis

Descriptive statistics including mean, standard deviation (SD), and range were computed for TL, DNF, and FI stratified by side. Shapiro–Wilk testing confirmed normality of continuous variables. Right–left comparisons used independent-samples t-tests; effect sizes are reported as Cohen’s d (pooled SD method). 95% confidence intervals (95% CI) for mean differences were computed using Welch’s t-distribution. Categorical variables were compared using Fisher’s exact test with Bonferroni correction for multiple surface comparisons (threshold *p* < 0.017). Wilson score 95% CIs are reported for proportions. No formal a priori power calculation was performed; this study is descriptive and the sample represents the complete available institutional collection meeting inclusion criteria. With *n* = 121 and α = 0.05 (two-sided), the study had 80% power to detect a bilateral difference in FI of ± 4.1% points, in TL of ± 5.7 mm, and in DNF of ± 6.1 mm. All analyses were performed using JAMOVI v2.3 (The JAMOVI Project, Sydney, Australia).

## Results

### Observer reliability

Inter- and intra-observer ICC values were excellent for both TL and DNF: TL inter-observer 0.96 (95% CI 0.92–0.98), DNF inter-observer 0.94 (95% CI 0.89–0.97); TL intra-observer 0.97 (95% CI 0.94–0.99), DNF intra-observer 0.95 (95% CI 0.91–0.98). Full data collection proceeded following confirmation of ICC ≥ 0.90 for both parameters.

### Number of nutrient foramina

Of 121 clavicles, 114 (94.2%) bore at least one nutrient foramen. Single foramina were most common (93 clavicles; 76.9%; 95% CI 68.6–83.5%), followed by double (13; 10.7%), triple (5; 4.1%), and four foramina (3; 2.5%); all three four-foramen clavicles were right-sided (Fig. 4). Seven clavicles (5.8%; 95% CI 2.8–11.5%) had no identifiable foramen. A total of 146 foramina were identified. No statistically significant side-to-side difference was detected (Fisher’s exact, all *p* > 0.05; Table 1).

**Table 1:** Nutrient foramen number distribution

Number of Nutrient Foramina	Right (*n* = 60) n (%)	Left (*n* = 61) n (%)	Total (*n* = 121) n (%; 95% CI)	*p* value†
No foramen	3 (5.0%)	4 (6.6%)	7 (5.8%; 2.8–11.5%)	0.696
Single foramen	47 (78.3%)	46 (75.4%)	93 (76.9%; 68.6–83.5%)	0.724
Double foramina	6 (10.0%)	7 (11.5%)	13 (10.7%; 6.4–17.5%)	0.796
Triple foramina	1 (1.7%)	4 (6.6%)	5 (4.1%; 1.8–9.3%)	0.171
Four foramina	3 (5.0%)	–	3 (2.5%; 0.8–7.0%)	–‡
Total foramina	74	72	146	–

†Fisher’s exact test right vs. left; ‡Expected cell frequency < 5, descriptive only. 95% CIs calculated using Wilson score method. Values n (%; 95% CI)

### Surface distribution

**Fig. 3:**
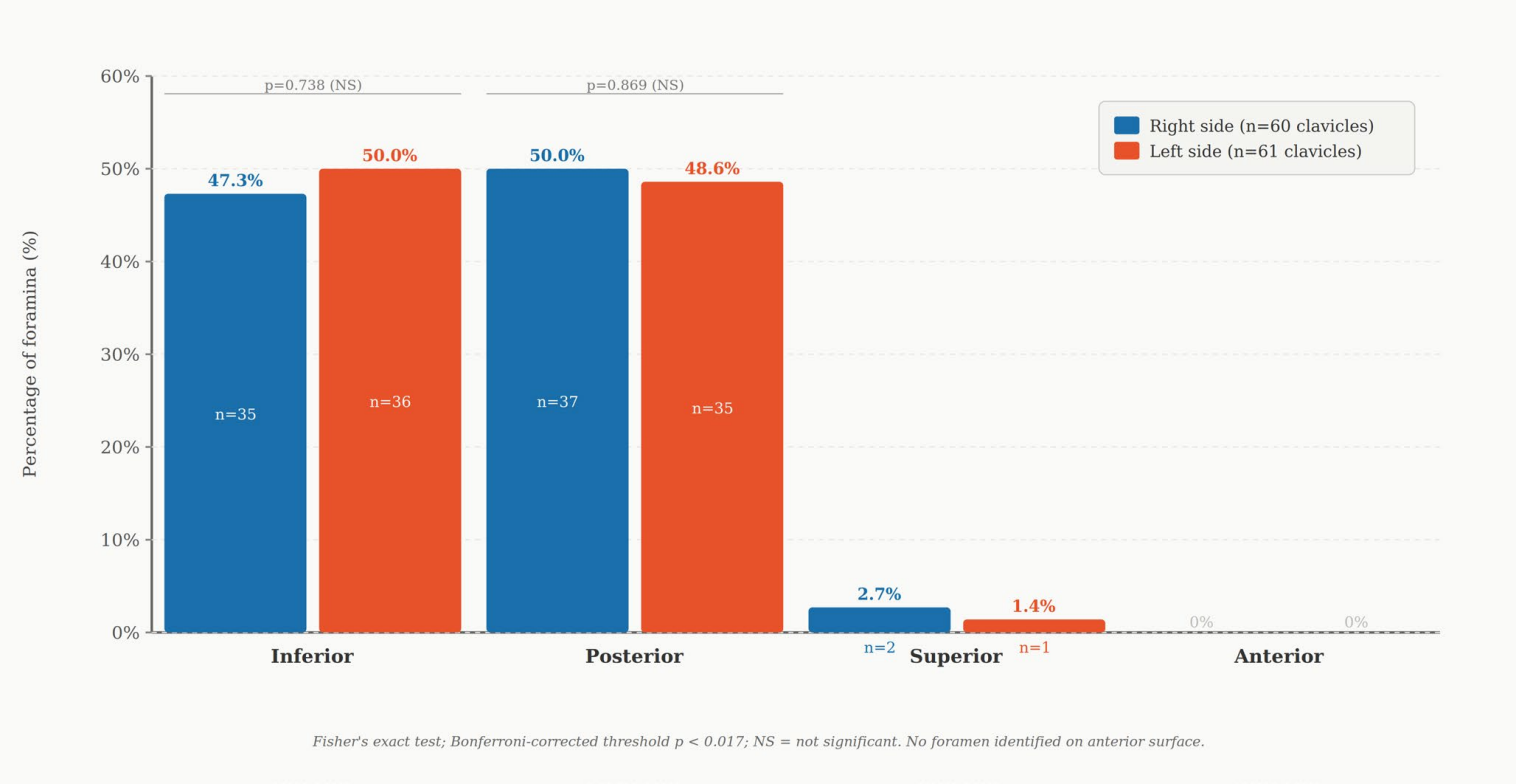
Grouped bar chart comparing surface distribution of clavicular nutrient foramina between right (*n* = 74 foramina) and left (*n* = 72 foramina) sides. Near-equal inferior and posterior distribution is demonstrated on both sides. No foramen was identified anteriorly. Fisher’s exact test; Bonferroni-corrected threshold *p* < 0.017; NS not significant

Surface analysis of all 146 foramina revealed near-equal distribution between the posterior (72; 49.3%; 95% CI 41.3–57.3%) and inferior (71; 48.6%; 95% CI 40.7–56.7%) surfaces (Fig. 3). Superior foramina were uncommon (3; 2.1%; 95% CI 0.7–5.9%); none were identified anteriorly (0.0%; 95% CI 0.0–2.6%). No significant laterality difference was detected after Bonferroni correction (Fisher’s exact, all *p* > 0.017; Table 2).

**Table 2:** Surface-wise distribution of 146 nutrient foramina in 121 clavicles

Surface	Right (*n* = 74) n (%)	Left (*n* = 72) n (%)	Total (*n* = 146) n (%; 95% CI)	*p* value†
Inferior	35 (47.3%)	36 (50.0%)	71 (48.6%; 40.7–56.7%)	0.738
Posterior	37 (50.0%)	35 (48.6%)	72 (49.3%; 41.3–57.3%)	0.869
Superior	2 (2.7%)	1 (1.4%)	3 (2.1%; 0.7–5.9%)	0.554
Anterior	0 (0.0%)	0 (0.0%)	0 (0.0%; 0.0–2.6%)	–
Total	74 (100%)	72 (100%)	146 (100%)	–

†Fisher’s exact test, Bonferroni-corrected threshold *p* < 0.017. 95% CIs calculated using Wilson score method. Values n (%; 95% CI)

### Regional localisation and morphometric measurements

Segment analysis restricted to 114 foraminated clavicles confirmed middle-third predominance in 112 (98.2%): 55 of 57 right-sided (96.5%) and all 57 left-sided (100.0%). Two right-sided clavicles had medial-third foramina (FI ≥ 0% and < 33%); none were lateral (*p* = 0.493). All 146 foramina were assigned to a single segment; no foramen spanned two FI-defined zones. In all cases, foramina co-occurring on multi-foramen clavicles were located within the same segment (middle third).

Mean TL was 138.5 ± 11.3 mm; left clavicles were significantly longer than right (141.0 ± 10.4 vs. 136.0 ± 11.9 mm; t(116.4) = 2.70, *p* = 0.008; Cohen’s d = 0.45; 95% CI for difference [− 9.0, − 1.0 mm]). Mean DNF was 72.1 ± 12.1 mm (right 70.6 ± 12.5, left 73.7 ± 11.4; t(117.6) = 1.69, *p* = 0.091; Cohen’s d = 0.26; 95% CI [− 7.4, 1.2 mm]). Mean FI was 52.1 ± 7.9% (right 51.9 ± 8.3%, left 52.3 ± 7.6%; t(117.7) = 0.35, *p* = 0.728; Cohen’s d = 0.05; 95% CI [− 3.3, 2.5%]). Full data are presented in Table 3.

**Table 3:** Regional localisation and morphometric measurements of clavicular nutrient foramina

Parameter	Right	Left	Total	*p* value
Segment distribution (n = 114 foraminated clavicles)*				
Medial third (FI ≥ 0% to < 33%)	2 (3.5%)	0 (0.0%)	2 (1.8%)	0.493†
Middle third (FI ≥ 33% to ≤66%)	55 (96.5%)	57 (100.0%)	112 (98.2%)	0.493†
Lateral third (FI > 66% to ≤100%)	0 (0.0%)	0 (0.0%)	0 (0.0%)	–
**Morphometric data (all 121 clavicles)**				
Mean TL (mm) ± SD	136.0 ± 11.9	141.0 ± 10.4	138.5 ± 11.3	0.008‡
95% CI for difference (right−left)	–	–	[− 9.0, − 1.0 mm]	–
Cohen’s d	–	–	0.45 (small)	–
TL range (mm)	111–158	120–161	111–161	–
Mean DNF (mm) ± SD	70.6 ± 12.5	73.7 ± 11.4	72.1 ± 12.1	0.091‡
95% CI for difference (right−left)	–	–	[− 7.4, 1.2 mm]	–
Cohen’s d	–	–	0.26 (small)	–
DNF range (mm)	20.0–96.0	43.0–99.0	20.0–99.0	–
Mean FI (%) ± SD	51.9 ± 8.3	52.3 ± 7.6	52.1 ± 7.9	0.728‡
95% CI for difference (right−left)	–	–	[− 3.3, 2.5%]	–
Cohen’s d	–	–	0.05 (negligible)	–
FI range (%)	34.6–70.8	36.1–68.4	34.6–70.8	–

*Segment analysis restricted to 114 foraminated clavicles. †Fisher’s exact test. ‡Independent-samples t-test (Welch). TL total clavicular length, DNF distance of nutrient foramen from sternal end, FI foraminal index, SD standard deviation, CI confidence interval. Values n (%) or mean ± SD

**Fig. 4:**
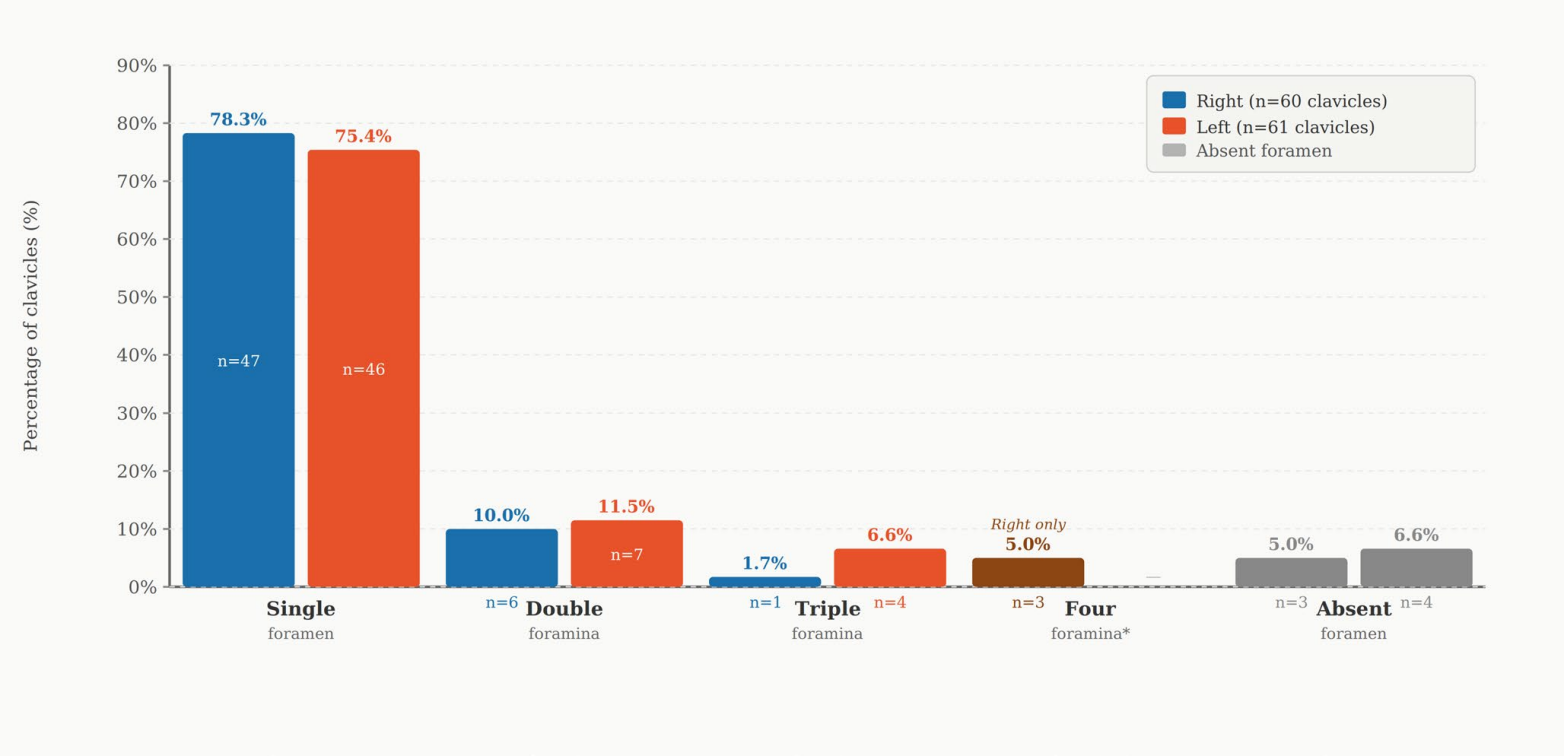
Grouped bar chart showing nutrient foramen number configuration in right (*n* = 60) and left (*n* = 61) clavicles. Single foramina were most prevalent on both sides. The four-foramen configuration was observed exclusively in right-sided clavicles (*n* = 3, 5.0%). Fisher’s exact test: all *p* > 0.05. ∗Four-foramen configuration: right side only

## Discussion

This study provides a methodologically transparent morphometric characterisation of clavicular nutrient foramina in 121 dry human clavicles — the largest Indian institutional series to date. As the present study is observational and based on dry cadaveric bone, all surgical inferences are presented as potential implications informed by the published literature, not as practice recommendations derived from the current data. Four principal findings emerge: (i) single foramina predominate (76.9%); (ii) foramina are distributed in near-equal proportions between the inferior and posterior surfaces, a pattern diverging from the posterior predominance of most European and Turkish series; (iii) the middle third is the near-universal segment (98.2%); and (iv) bilateral symmetry of proportional foramen position is preserved despite a statistically significant absolute length difference between sides.

### Foramen count and absent foramina

The single-foramen prevalence of 76.9% aligns with Turkish series [21, 22] and Ejlersen’s meta-analytic data [6], supporting methodological reproducibility when fine-wire patency confirmation is applied. The higher single-foramen rate compared to Murlimanju et al. [14] (38.5%) reflects identification methodology: fine-wire confirmation excludes non-patent vascular pits included by some earlier studies [6]. The 5.8% absent-foramen prevalence is within the published range; in such clavicles medullary perfusion depends entirely on periosteal vessels [11], which has been noted in the literature as a consideration during subperiosteal dissection [12]. The three right-sided four-foramen clavicles (2.5%; Fig. 4) extend the midshaft zone over which foramen-related considerations apply.

### Surface distribution: a potentially relevant finding

The near-equal inferior (48.6%) and posterior (49.3%) surface distribution (Table 2; Fig. 3) diverges from the posterior predominance (52–82%) of European, Turkish, and Bangladeshi series [2, 12, 21, 22]. This pattern has also been reported by Murlimanju et al. [14] from a different South Indian institution, and by Dakshayani and Shivanal [5] in a Central Indian series. A synthesis of all available Indian surface distribution data (Table 4) reveals that inferior surface predominance or parity is more consistently reported in Indian series than in European or Turkish data. Posterior predominance within Indian series is reported by Aggarwal and Ghorai [1] (North Indian) and Ratnesh et al. [18] (Bihar), suggesting that the pattern may vary within India. The present study does not hypothesise that Indian regional subgroups represent biologically distinct populations; the regional subgrouping in Table 4 is organisational. The convergence of inferior-surface findings in two independent South Indian series is noted as a potentially relevant observation requiring prospective investigation with documented demographic data before population-level conclusions can be drawn.

The anatomical basis for inferior-surface foramina has been described by Knudsen et al. [11] in a cadaveric vascular injection study, which demonstrated that the suprascapular nutrient branch can descend across the inferior cortex before piercing the diaphysis. This is consistent with published variation in suprascapular artery branching patterns [4]. Published literature on axillary artery variants has been proposed as a general consideration for surgeons working in this region [7, 12]; the present data add surface distribution information that may be relevant to planning in this population, while acknowledging that direct clinical validation is required.

### Bilateral TL asymmetry

The statistically significant difference in mean TL (left > right by 5.0 mm; *p* = 0.008; Cohen’s d = 0.45, small effect) is consistent with published reports of clavicular length asymmetry attributed to limb dominance-related remodelling [20]. Yarar et al. [22] and Vatansever and Demiryürek [21] both reported that FI did not differ significantly between sexes or sides in their series; the present finding of non-significant FI laterality difference (*p* = 0.728; d = 0.05, negligible) is therefore consistent with the international literature. The absence of hand dominance data in the present series prevents formal confirmation of the association between TL asymmetry and dominance. The practical implication — that the proportional position of the foramen is bilaterally symmetric despite absolute length differences — supports the use of contralateral clavicle radiographs as a reference for length restoration in unilateral fracture fixation [3], irrespective of side-to-side length difference.

### Middle-third predominance

The 98.2% middle-third predominance and mean FI of 52.1% are consistent with Ejlersen’s pooled range (36.31–61.03%) [6] and with Turkish [21, 22], German [12], and Bangladeshi [2] comparator series (Table 4). These findings are consistent across dry bone, cadaveric, and radiological study designs, suggesting that the middle-third localisation is a robust anatomical feature. Dry bone studies allow large samples and direct surface measurement but cannot assess arterial patency or haemodynamic function. Cadaveric injection studies [7, 11] directly visualise the nutrient artery and its cortical entry but are limited in sample size. Radiological approaches allow in vivo assessment but may underdetect small foramina due to resolution constraints. The present dry bone approach is appropriate for the study’s descriptive objectives and is consistent with the majority of published institutional series (Table 4).

### Limitations

The retrospective design and absence of documented sex, age, and hand dominance data preclude stratified analyses — a limitation shared by all eight comparator institutional dry bone studies in Table 4 [1, 2, 5, 12, 14, 18, 21, 22]. In the context of the statistically significant bilateral TL difference (*p* = 0.008), the absence of hand dominance data means we cannot confirm whether the pattern reflects expected right-side remodelling associated with right-hand dominance. Yarar et al. [22] reported no significant sex-based FI difference in their Turkish series; the present lack of sex data therefore likely has limited impact on the FI findings specifically. Although *n* = 121 represents the largest published Indian institutional dry bone clavicular series, the sample remains modest relative to pooled international data (Ejlersen: *n* = 3,760 [6]); prevalence estimates for rare configurations carry wide confidence intervals and should not be over-interpreted. Generalisation to other Indian regions or to the broader South Asian population is unknown. Foramen orifice diameter and canal direction were not assessed; several published series have reported these parameters [14, 22], and their inclusion in future studies would enable more complete morphometric characterisation. Fine-wire probing cannot visualise arterial entry angle, canal diameter, or haemodynamic significance.

## Conclusion

This institutional dry bone study identifies a near-equal distribution of clavicular nutrient foramina between the inferior and posterior surfaces — a pattern consistently observed in two independent South Indian series and distinct from the posterior predominance reported in most European and Turkish studies. Middle-third localisation and bilateral symmetry of proportional foramen position are confirmed. These findings contribute population-specific morphometric data and suggest that both midshaft cortical surfaces may warrant awareness during clavicular surgical approaches in this population; formal clinical validation is required before operative recommendations can be made.

### Comparative data

See Table 4.

**Table 4:** Published studies on clavicular nutrient foramina

Author (Year)	Population	Method	*n*	Single NF (%)	Surface	Mean FI (%)	Segment
De Garis & Swartley (1928)	American	Cadaver	Various	NR	Variable	NR	Middle
Nordqvist & Petersson (1994) [16]	Swedish	Radiological	782	NR	NR	NR	NR
Murlimanju et al. (2011) [14]	South Indian	Dry bone	52	38.5	Inf/Post	NR	Middle (92.3%)
Murlimanju et al. (2011) [15]	South Indian	Dry bone	52	NR	NR	NR	Middle
Haładaj et al. (2015) [NR]	Polish	Cadaver	1000	NR	NR	NR	Middle
Leschinger et al. (2019) [12]	German	Cadaver	51	66.7	Posterior	~ 54.9	Middle
Vatansever & Demiryürek (2020) [21]	Turkish	Dry bone	130	~ 80	Posterior	51.3 ± 7.7	Middle
Aggarwal & Ghorai (2021) [1]	North Indian	Dry bone	79	55.7	Posterior	43.8 ± 11.9	Middle (67.5%)
Dakshayani & Shivanal (2021) [5]	South Indian	Dry bone	50	76.0	NR	NR	Middle
Ejlersen (2023) [6] (SR/MA)	Pooled (33 studies)	Mixed	3760	NR	Posterior	36.3–61.0	Middle
Ara et al. (2024) [6]	Bangladeshi	Dry bone	150	~ 61	Posterior	~ 47.5	Middle
Mazengenya et al. (2025) [13]	South African	Cadaver	84	NR	NR	NR	Middle
Yarar et al. (2025) [22]	Turkish	Dry bone	NR	~ 76	Posterior	~ 50.2	Middle
Present study (2025)	South Indian	Dry bone	121	76.9	Inf≈Post	52.1 ± 7.9	Middle (98.2%)

NF nutrient foramen, FI foraminal index, NR not reported, SR/MA systematic review and meta-analysis, Inf inferior, Post posterior. †Approximate values estimated from reported data in the cited study (see text). Present study: institutional collection, South India; unpaired dry bones of unknown age and sex

## Data Availability

No datasets were generated or analysed during the current study.
